# Nephroprotective Effect of L-Carvone on a Mouse Model of LPS-Induced Sepsis-Associated Renal Injury via Regulation of TLR4/NF-κB/AP-1/IRF-3 and Nrf2/iNOS Molecular Signaling Cascades

**DOI:** 10.1016/j.jtumed.2025.12.008

**Published:** 2026-01-12

**Authors:** Saja M. Shareef, Sarmed H. Kathem, Hayder Ridha-Salman

**Affiliations:** a Department of Pharmacology and Toxicology, College of Pharmacy, Al-Esraa University, Baghdad, Iraq; b Department of Pharmacology and Toxicology, College of Pharmacy, University of Baghdad, Baghdad, Iraq; c Al-Mustaqbal University, College of Pharmacy, Hillah, Babylon, Iraq

**Keywords:** L-carvone, Monoterpenoids, NF-κB, Renal failure, Sepsis, -كارفون, االإنتان, القصور الكلوي, NF-κB, التربينات الأحادية

## Abstract

**Background:**

LPS-evoked endotoxemia triggers systemic inflammation and can cause multi-organ damage, with the kidneys being particularly vulnerable. L-carvone is a natural monoterpene with strong antimicrobial, cytoprotective, and immunomodulatory benefits.

**Objective:**

This study was aimed at exploring the influence of L-carvone on LPS-aggravated sepsis-associated renal impairment in mice.

**Methods:**

A total of 32 male albino-type mice were randomly divided into six groups: untreated control group, sepsis model group (LPS 10 mg/kg single dose), vehicle group (oral corn oil for 5 days before LPS injection), and three intervention groups orally pre-treated with low (25), moderate (50), or high (100) mg/kg doses of L-carvone for 5 continuous days before LPS challenge.

**Results:**

L-carvone markedly decreased levels of KIM-1, BUN, and creatinine, and reversed renal histological aberrations. It downregulated TLR4, NF-κB, AP-1, IRF-3, and iNOS renal expression, while upregulating Nrf2 transcription in a dose-dependent manner, thus decreasing interleukin (IL)-1β and TNF-α concentrations. L-carvone further ameliorated pro-apoptotic Bax levels, increased anti-apoptotic Bcl-2, inhibited MDA production, and enhanced SOD activity.

**Conclusion:**

L-carvone effectively mitigates sepsis-related renal impairment by counteracting inflammatory, oxidative, and apoptotic mechanisms, thus supporting its translational therapeutic promise.

## الملخص

**أهداف البحث:**

يُعد الإنتان من الحالات السريرية الخطيرة الناتجة عن استجابة مناعية غير منضبطة، تؤدي إلى التهاب جهازي واسع النطاق وإصابة متعددة للأعضاء، وتُعد الكلى من أكثر الأعضاء تأثرًا. ويلعب التسمم الداخلي المحفَّز بالذيفان الشحمي (LPS) دورًا رئيسيًا في تطور القصور الكلوي المصاحب للإنتان. ويُعد مركب L-كارفون أحد المركبات الطبيعية من فئة التربينات الأحادية، ويتمتع بخصائص مضادة للالتهاب والأكسدة ومعدِّلة للمناعة. هدفت هذه الدراسة إلى تقييم التأثير الوقائي لمركب L-كارفون ضد القصور الكلوي المصاحب للإنتان في نموذج حيواني.

**طرق البحث:**

أُجريت دراسة تجريبية على 32 فأرًا ذكريًا من نوع BALB/c، وُزِّعت عشوائيًا إلى ست مجموعات: مجموعة ضابطة غير معالجة، مجموعة الإنتان حُقنت بجرعة واحدة من LPS بتركيز 10 ملغم/كغم، مجموعة الحامل (زيت الذرة)، وثلاث مجموعات عولجت مسبقًا فمويًا بمركب L-كارفون بجرعات 25 و50 و100 ملغم/كغم لمدة خمسة أيام قبل تحريض الإنتان. جرى تقييم مؤشرات وظائف الكلى، والتغيرات النسيجية، والتعبير الجيني لعوامل الالتهاب والإجهاد التأكسدي، إضافة إلى مؤشرات الموت الخلوي المبرمج.

**النتائج:**

أظهر العلاج بمركب L-كارفون انخفاضًا ذا دلالة في مستويات نيتروجين يوريا الدم، والكرياتينين، وجزيء إصابة الكلى-1، مع تحسُّن ملحوظ في البنية النسيجية للأنسجة الكلوية مقارنة بمجموعة الإنتان. كما أدى L-كارفون إلى تثبيط التعبير الجيني لمستقبل TLR4 وعوامل النسخ الالتهابية NF-κB وAP-1 وIRF-3 وإنزيم iNOS، بالتزامن مع زيادة التعبير الجيني لعامل Nrf2، مما انعكس في انخفاض مستويات السيتوكينات الالتهابية IL-1β وTNF-α. إضافةً إلى ذلك، انخفض التعبير عن المؤشر المحفِّز للاستماتة Bax وارتفع التعبير عن البروتين المضاد للاستماتة Bcl-2، مع تقليل نواتج الأكسدة الدهنية وزيادة نشاط إنزيم سوبر أوكسيد ديسميوتاز، وذلك بصورة تعتمد على الجرعة.

**الاستنتاج:**

يُسهم مركب L-كارفون في التخفيف من القصور الكلوي المصاحب للإنتان من خلال تثبيط الاستجابة الالتهابية، والحد من الإجهاد التأكسدي، وتنظيم مسارات الموت الخلوي المبرمج، مما يدعم إمكاناته العلاجية كمركب واعد في تدبير الإنتان المصحوب بإصابة كلوية.

## Introduction

Sepsis involves dysregulation of the immunological defense mechanisms in response to infection and severe systemic inflammation that can rapidly progress to multi-organ failure and death.^1^^,^^2^ Among the affected organs, the kidneys are particularly vulnerable. Sepsis-associated acute kidney injury (SA-AKI) is an ongoing and major sequela that develops in as many as 50 % of patients with critical infections or circulatory collapse. SA-AKI not only increases morbidity and mortality but also extends hospitalization stays and leads to substantial healthcare costs; therefore, new strategies are needed to prevent or treat AKI.^3^^,^^4^ (see Table 1)

**Table 1:** Amplification primers for target genes.

Gene	Primer direction	Sequences (5ʹ→3ʹ direction)
GAPDH	Forward Reverse	CGGGTTCCTATAAATACGGACTG CCAATACGGCCAAATCCGTTC
NF-κB	Forward Reverse	AAGACAAGGAGCAGGACATG AGCAACATCTTCACATCCC
TLR4	Forward Reverse	TCCCTGCATAGAGGTAGTTCC TCAAGGGGTTGAAGCTCAGA
Kim-1	Forward Reverse	GGCTCTCTCCTAACTGGTCA CCACCACCCCCTTTACTTCC
AP-1	Forward Reverse	AG GCTGCAGGATGATGCGAT TTCTAGCCAGGACGACTTGC
IRF3	Forward Reverse	CAATTCCTCCCCTGGCTAGA GGGATCCTGAACCTCGTTCG
IL-1β	Forward Reverse	TGCCACCTTTTGACAGTGATG TGATGTGCTGCTGCGAGATT
iNOS	Forward Reverse	GGTGAAGGGACTGAGCTGTT ACGTTCTCCGTTCTCTTGCAG
Nrf2	Forward Reverse	CAGCATAGAGCAGGACATGGAG GAACAGCGGTAGTATCAGCCAG

The underlying etiology of SA-AKI is intricate and multifactorial, involving hemodynamic instability, microvascular abnormalities, diminished intrarenal circulation, endothelium deterioration, oxidative stress, imbalanced immunity mechanisms, inflammatory cell invasion, and tubular apoptosis.^5^^,^^6^ Substantial immune system triggering has been found to lead to cell-mediated and antibody-dependent processes.^1^^,^^7^ At the core of these processes is an extensive inflammatory response to microbial-derived components, particularly the lipopolysaccharide (LPS) in Gram-negative bacterial walls.^8^^,^^9^ LPS, a prototypical initiator of systemic immune responses, engages with host cell surface receptor assemblies, including Toll-like receptor 4 (TLR4) and CD14, and subsequently orchestrates downstream inflammatory signaling events.^10^^,^^11^ The stimulation of TLR4 generated on renal tubular epithelial cells, endothelial lining, and various immune cell groups triggers two key downstream signaling networks: an MyD88-driven cascade that initiates early pro-inflammatory gene regulation and an acute TRIF-driven route that primarily regulates interferon-β formation and late-phase inflammatory responses.^12^ Both pathways culminate in stimulation of the transcription factors nuclear factor-kappa B (NF-κB), activator protein-1 (AP-1), and interferon regulatory factor-3 (IRF-3), which in turn facilitate the synthesis of proinflammatory substances including interleukin (IL)-1β, IL-6, and tumor necrosis factor-alpha (TNF)-α.^11^^,^^13^^,^^14^ A consequent cytokine storm drives endothelial injury, leukocyte infiltration, and oxidative stress, and leads to tubular necrosis, failed glomerular filtration, and elevation in indicators of renal impairment (blood urea nitrogen [BUN], creatinine, and kidney injury molecule-1 [KIM-1]).^15^^,^^16^ NF-κB promotes the ROS-driven breakdown of nucleic acids, fats, and protein molecules, and leads to cell death and tissue destruction.^17^^,^^18^

Even with improvements in supportive care, the management of SA-AKI is restricted to hemodynamic support, fluid resuscitation, vasopressors, and renal replacement therapy. Although pharmacologic strategies that decrease inflammation and oxidative stress have been found to be effective in preclinical models, their clinical translation has been limited by low efficacy, off-target effects, or toxicity.^19^^,^^20^ Consequently, naturally occurring phytosubstances with anti-inflammatory and antioxidative qualities have been studied as adjunctive treatments for sepsis and organ injury.^21^^,^^22^

Phytocompounds are being rigorously evaluated as potential therapies for a range of conditions including hepato-renal injuries.^23^^,^^24^ Terpenoids, a diverse group of plant-extracted substances created from isoprene fragments, demonstrate a broad spectrum of medicinal activities, notably free radical scavenging, anti-bacterial, anti-inflammatory, and cancer-combating capabilities, and therefore are valuable in multiple clinical protocols.^25^, ^26^, ^27^ Those constituents have shown promise in renoprotection and potential applications in the treatment of kidney-related conditions.^28^, ^29^, ^30^

L-carvone, a natural monoterpene ketone, is the principal component of essential oils obtained from plants including caraway (*Carum carvi*) and spearmint (*Mentha spicata*).^31^ It comprises a pair of double enantiomers, (R)- and (S)-carvone, which have distinct medicinal actions, notably antimicrobial, antioxidant, anti-inflammatory, and cytoprotective attributes.^32^^,^^33^ The mechanism of action of L-carvone has been reported to involve altered levels of several inflammatory and oxidative stress-related molecular signaling, namely NF-κB, mitogen-activated protein kinases (MAPKs), and inducible nitric oxide synthase (iNOS), and to ultimately decrease the release of pro-inflammatory cytokines and tissue damage in multiple experimental models.^34^ In addition, L-carvone shows organoprotective actions in hepatic, myocardial, and neuronal injuries, primarily through its ability to mitigate oxidative stress and inflammatory responses.^35^^,^^36^ Consequently, it might be used to treat the nephrotoxicity associated with systemic inflammation.

Given that TLR4-driven, MyD88-related, and TRIF-dependent pathways are critically involved in the occurrence of SA-AKI, and that transcription factors, particularly NF-κB, AP-1, and IRF-3, are primary regulators of renal damage, targeting these pathways might have therapeutic promise.^3^^,^^37^ The potential of L-carvone to control inflammatory mediators and transcriptional regulators might enable a multi-target one-shot approach to ameliorate renal inflammation, tubular injury, and dysfunction in sepsis.

Therefore, this study was aimed at determining L-carvone's effects against sepsis-exacerbated renal impairment in mice and exploring its underlying molecular effects beyond its established pharmacological actions.

## Materials and Methods

### Ingredients and indicators

Sigma–Aldrich, Inc. provided L-carvone and LPS. The ELISA kits were sourced from Shanghai, China. Macrogen Corporation, Seoul/South Korea, donated primer sets for AP-1, IRF3, KIM-1, IL-1β, NF-κB, INOS, and Nrf2. Other bio-indicators and supplies used herein were sourced from reputable companies.

### Methodological setup

**Figure 1:**
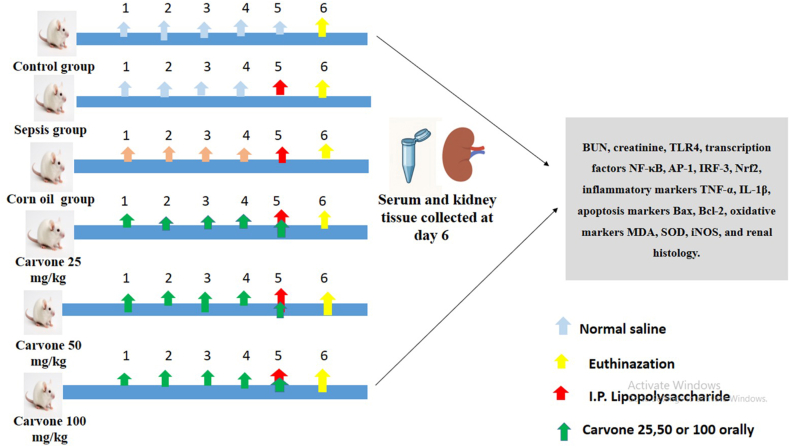
Schematic illustration of experimental design.

Male albino-type BALB/c (n = 36) 8–10 weeks old and weighing 20–30 g were used for physiological consistency and were randomly assigned to one of six groups. Animals were selected within specific age and weight ranges to decrease variation in pharmacokinetic and immunogenic responses. Only male mice were used, to limit the effects of sex hormones on inflammatory/biochemical responses, because female estrogen and progesterone levels can influence sepsis-associated pathophysiology consequences.^38^ Figure 1 shows a graphical illustration of the experimental protocol. The animal groups were as follows.

#### Non-treated controls

Mice received intraperitoneal (IP) 100 μL 0.9 % sodium chloride solution.

#### LPS/sepsis model

Mice were injected with a single IP LPS 10 mg/kg dose.^39^ Corn oil/vehicle: Mice received 100 μL corn oil via gastric lavage for 5 successive days before IP administration of 10 mg/kg of LPS on the 5th day. L-carvone-25 (low dosage): Mice received a low dosage of 25 mg/kg of L-carvone by gastric lavage for 5 successive days, and subsequent IP administration of 10 mg/kg of LPS on the 5th day.^32^ L-carvone-50 (moderate dosage): Mice received a moderate dosage of 50 mg/kg of L-carvone via gastric lavage for 5 successive days, and subsequent IP administration of 10 mg/kg of LPS on the 5th day.^32^ L-carvone-100 (high dosage): Mice received a high dosage of 100 mg/kg of L-carvone via gastric lavage for 5 successive days, and IP administration of 10 mg/kg of LPS on the 5th day.^32^

The LPS dose (10 mg/kg IP) used herein is recognized as the standard model for endotoxin-driven renal sepsis.^39^^,^^40^ This dose elicits substantial renal damage, tubular necrosis, and amplification of inflammatory cytokines without excessive mortality.^18^ Hence, it provides a good representation of the pathophysiologic course of septic AKI and is suitable for assessing treatment choices. The doses of L-carvone (25 mg/kg, 50 mg/kg, and 100 mg/kg) were selected according to prior pharmacological research demonstrating anti-inflammatory, antioxidative, and cytoprotective benefits and reasonable safety profiles at these doses.^32^^,^^35^

After completion of the trial, all mice were euthanized with a ketamine (80 mg/kg) and xylazine (8 mg/kg) anesthetic protocol 24 h after receiving the final dose of the investigated compounds, and tissue samples were promptly collected for analyses.^41^^,^^42^

### Biochemical analysis

Mouse blood specimens were obtained through retro-orbital collection and centrifuged at 3000 rpm at 4 °C. Serum was collected and transferred into marked microcentrifuge tubes, and stored at −20 °C until analysis.^43^^,^^44^

### Histological analysis

After euthanasia, the left renal tissues were removed, washed in phosphate-buffered saline, and preserved in 10 % NBF. To avoid tissue distortion and ensure efficient water drainage, we progressively desiccated samples in a graded alcohol series for 120 min.^45^^,^^46^ The specimens were cleared with xylene to enhance visualization. Next, kidney samples were embedded in wax, sectioned into 5 μm slices, and treated with hematoxylin and eosin.^47^^,^^48^ A blinded pathologist inspected the fragments via light microscopy. Five randomly selected areas per slide were imaged at × 40 magnification through digital optical microscopy. A semi-quantitative scoring method was used to calculate tubular cell erosion, brush boundary loss, and tubular dilatation: 0, normal; 1, <25 %; 2, 25–50 %; 3, 50–75 %; and 4, >75.^49^

### RNA preparation and qRT-PCR analyses

Trans Generation Biotech (China) provided the single-step gDNA removal and cDNA Synthesis SuperMix, RNA extraction products, and a Top Green qPCR SuperMix for SYBR Green I.

### Protein quantification by ELISA

Briefly, 0.05 mL reference mixture and 0.04 mL of each specimen were added to the respective wells. Subsequently, 0.01 mL of biotin-tagged antibodies to TNF-α, Bax, Bcl-2, MDA, and SOD were loaded into the analyte wells, together with 0.05 mL streptavidin-HRP. The plates were gently mixed, sealed, and incubated at 37 °C for 60 min.^50^^,^^51^ After five washes with wash buffer, 300 μL wash solution was added to each well. Subsequently, 0.05 mL each of reagents A and B was added and incubated at 36 °C for 10 min. The reaction was stopped, thus causing the color to change from blue to yellow. Spectrophotometric measurements were estimated at a 450-nm wavelength with a multiwell-plate detector within 10 min, and concentrations of the measured markers were determined according to optical density readings.^52^^,^^53^

### Statistical analyses

The outcomes are reported as means ± standard error of the mean (SEM). Version 25 of the Statistical Package for the Social Sciences was used for statistical analysis. With one-way ANOVA, the same parameter was compared among groups and was followed by post-hoc analysis with Tukey's test. *P* > 0.05 was considered to indicate statistical significance.

## Results

### Influence of L-carvone on functional bio-indicators

**Figure 2:**
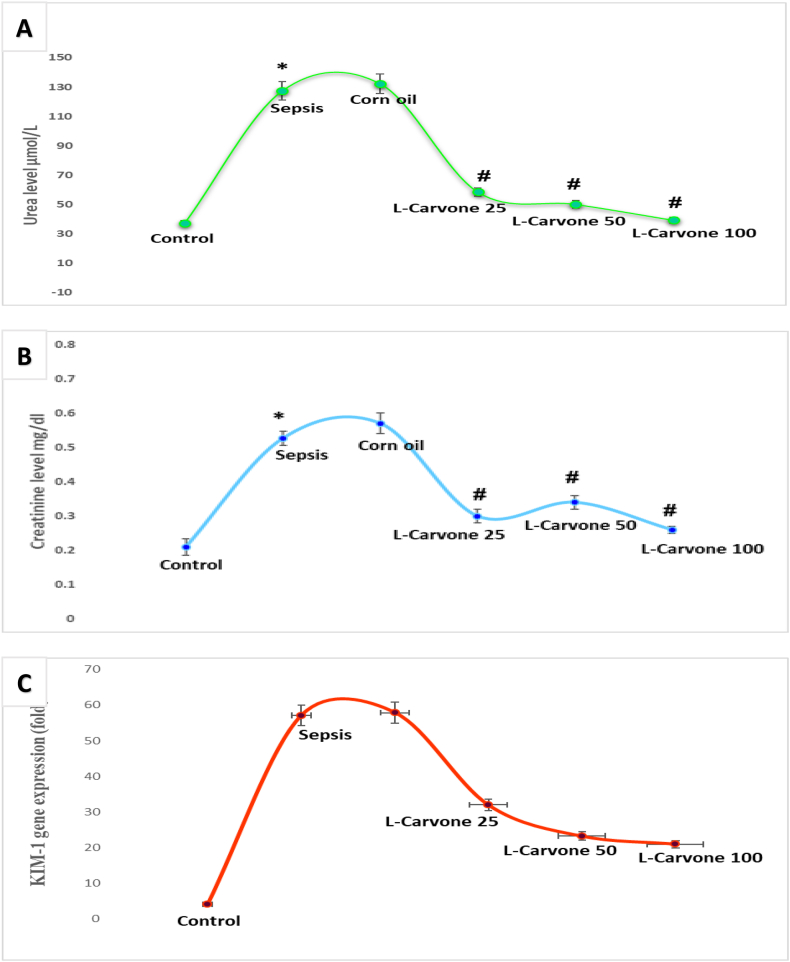
**A**. Influence of the investigated agents on urea levels in the serum. **B**. Influence of the investigated agents on creatinine levels in the serum. **C**. Influence of the investigated agents on renal levels of KIM-1. Results are means ± SEM; ∗P < 0.05 compared with control, #P < 0.05 compared with sepsis/induction.

Mice subjected to sepsis development via LPS administration showed compromised renal performance, as reflected by markedly greater BUN and creatinine content than observed in the untreated controls; these findings were predictive of renal impairment. In contrast, the marked mitigation of BUN and creatinine production in the intervention groups suggested that L-carvone had renoprotective properties (Figures 2A and 2B).

KIM-1 is a target-sensitive bio-indicator of renal compromise. The sepsis group showed significantly greater KIM-1 concentrations than the controls. Pre-intervention with L-carvone at low (25), moderate (50), or high (100) mg/kg doses led to significantly lower KIM-1 than observed in the sepsis group. Furthermore, the corn oil group showed non-significant differences with respect to the sepsis group (Figure 2C).

**Figure 3:**
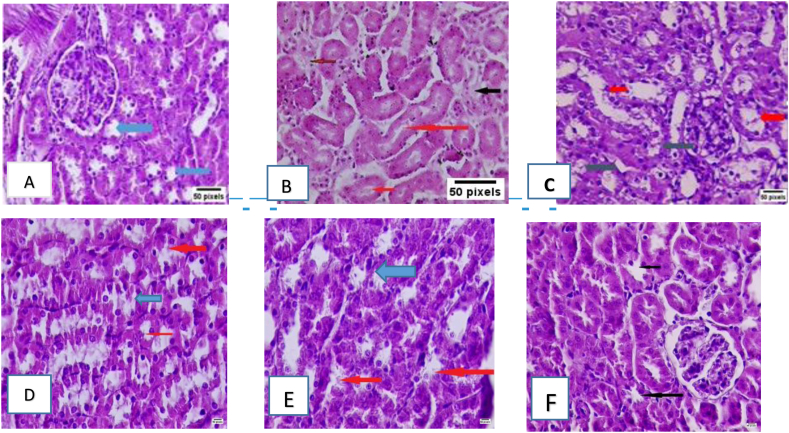
**Influence of the investigated agents on renal histology abnormalities.** Representative photomicrographs of renal slices from every group at a power of × 40, scale bar = 50 µm. The blue horizontal bars demonstrate undamaged proximal tubules, the black bars show sloughed tubular cells, and the red bars show brush boundary loss during tubular dilatation. Panel A is the untreated control, panel B is the LPS-evoked renal sepsis model, panel C is the vehicle control (corn oil), panel D is the L-carvone low dose group (25 mg/kg), panel E is the L-carvone moderate dose group (50 mg/kg), and panel F is the L-carvone high dose group (100 mg/kg).

The control group showed normal renal histology with mild glomerular aggregates, semi-normal interstitial cells, and an absence of inflammatory changes (Figure 3A). In the sepsis group, the kidneys showed notable tubular degeneration, dilatation, swelling of epithelial cells, cytoplasmic vacuolation, and focal necrosis of proximal tubular cells, thus indicating acute tubular injury accompanied by severe interstitial inflammatory cell infiltration (Figure 3B). These degenerative alterations indicated the inflammatory cytotoxicity generated by LPS-stimulated induction of the TLR4/NF-κB axis, which ultimately results in oxidative attack and cytokine-exacerbated tissue damage. In contrast to the untreated control group, the corn oil/vehicle group showed notable dilatations of the tubular cavity, accompanied by moderately diminished apical microvilli edges and nephronic detachment (Figure 3C). The L-carvone-treated (25 mg/kg) group displayed moderately ameliorated histological changes, with partially preserved tubular architecture, diminished epithelial degeneration, moderate inflammatory cell infiltrate, and slight restoration of the brush border (Figure 3D). Furthermore, in the L-carvone-treated group (50 mg/kg), a marked histological improvement was observed, with mild tubular architecture, moderately diminished epithelial degeneration, and mild infiltration of inflammatory cells (Figure 3E). More importantly, in the L-carvone-treated group (100 mg/kg), a highly substantial histological improvement was observed, with a virtually intact tubular appearance, loss of epithelial degeneration, and mild invasion of inflammatory cells (Figure 3F). These results validated a dose-dependent histological amelioration, in agreement with the biochemical data.

**Figure 4:**
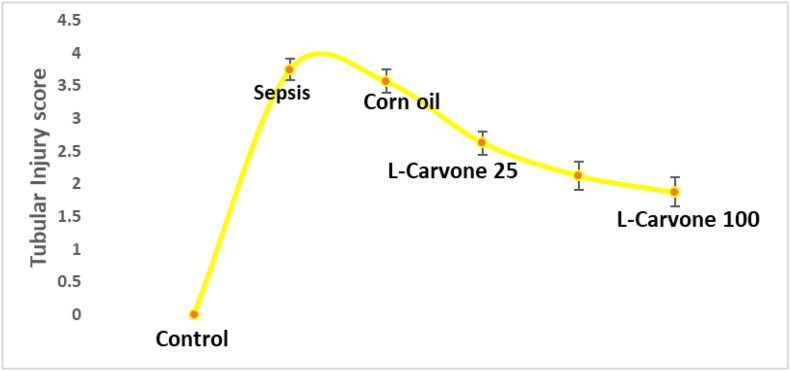
Semiquantitative estimation of tubular damage. Results are means ± SEM; ∗P < 0.05 compared with control, #P < 0.05 compared with sepsis/induction.

Analysis of tubular damage scores revealed that treatment with different doses of L-carvone, compared with the control, was associated with substantially lower tubular damage grades (Figure 4).

### Influence of L-carvone on TLR4, AP-1, and IRF3 expression

**Figure 5:**
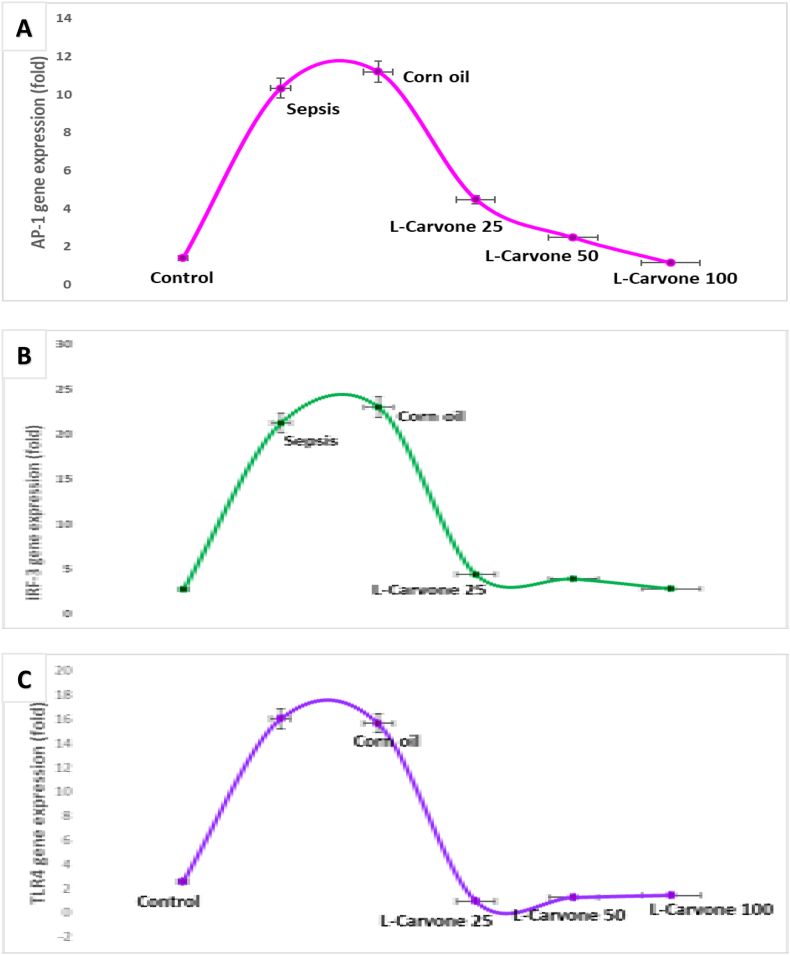
**A**. Influence of the investigated agents on mRNA TLR-4 expression. **B**. Influence of the investigated agents on AP-1 gene expression. **C**. Influence of carvone on IRF3 gene expression. Results are means ± SEM; ∗P < 0.05 compared with control, #P < 0.05 compared with sepsis/induction.

Sepsis model rats administered LPS showed substantially greater TLR4-mRNA transcription than non-treated controls. Animals treated with L-carvone at all three doses showed markedly lower TLR4 mRNA levels than observed in the sepsis model group (Figure 5A).

Moreover, AP-1 mRNA expression was markedly greater in the sepsis induction/model than the non-treated control. Paradoxically, the interventions with all L-carvone doses showed diminished AP-1 transcription. In addition, the corn oil group showed non-substantial differences with respect to the sepsis/induction model (Figure 5B).

The outcomes of IRF3 genetic expression (Figure 5C) indicated that LPS-exposed mice in the sepsis group showed markedly greater IRF3 gene expression than untreated controls. Meanwhile, the application of L-carvone in three doses resulted in significantly lower IRF3 levels than observed in the sepsis model. Meanwhile, corn oil produced non-significant changes with respect to the sepsis model.

### Influence of L-carvone on inflammation-associated responses

**Figure 6:**
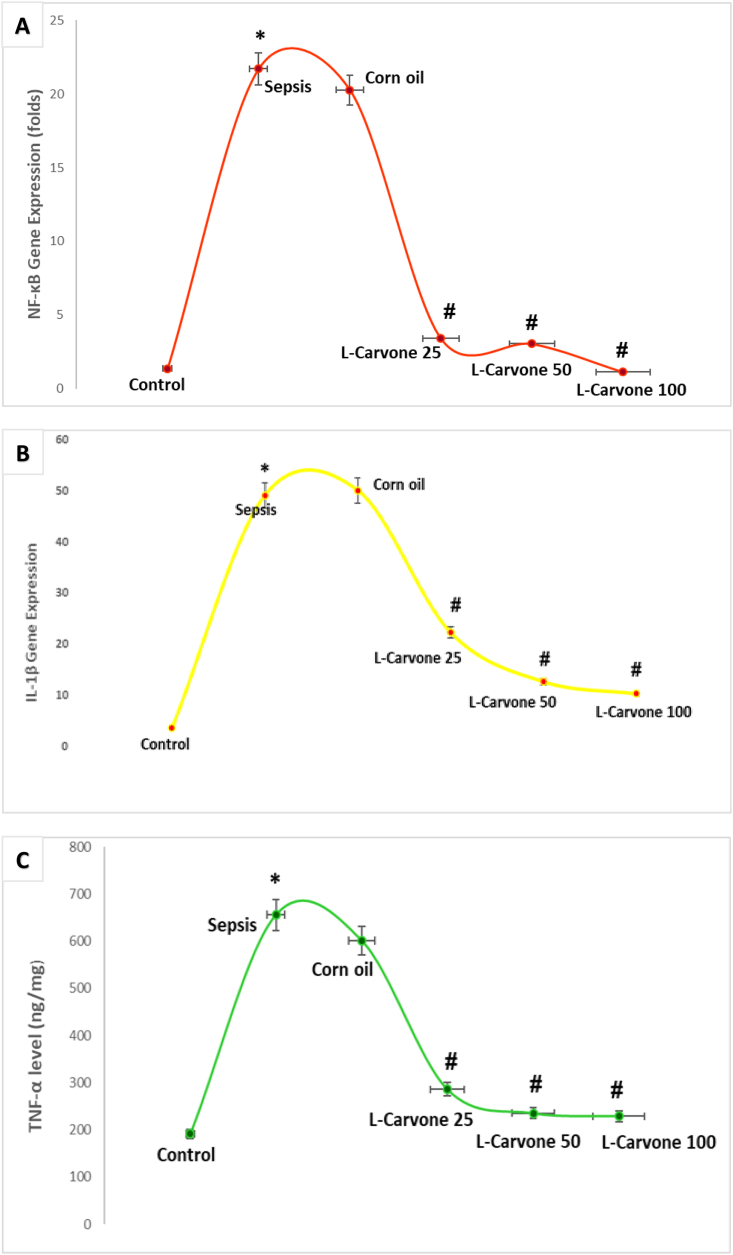
**Influence of the investigated agents on the expression of inflammation-related parameters (NF-κB expression, IL-1β expression, and TNF-α level) in sepsis-evoked kidney impairment. A.** Transcriptional profile of NF-κB. **B.** Transcriptional profile of IL-1β. **C.** ELISA profile of TNF-α. NF-κB and IL-1β are expressed as fold changes, whereas TNF-α is quantified in ng/L. Results are means ± SEM; ∗P < 0.05 compared with control, #P < 0.05 compared with sepsis/induction.

At 24 h after LPS injection, the expression of NF-κB mRNA was markedly greater in the sepsis/LPS-induction group than the untreated control group. Pre-treatment with L-carvone at all concentrations, in contrast to sepsis/LPS induction, markedly suppressed NF-κB transcription (Figure 6A). LPS-treated mice in the sepsis/induction model group had considerably greater expression of IL-1β and TNF-α within kidney tissues than untreated controls. Corn oil was used as a vehicle/carrier for L-carvone, and animals given oral corn oil for 5 successive days before LPS treatment showed no notable differences with respect to those in the sepsis model group. L-carvone therapy (at low, moderate, and high doses) led to markedly lower kidney IL-1β expression and TNF-α concentrations than observed after sepsis/LPS induction (Figures 6B and **6C**).

### Influence of the investigated agents on iNOS and Nrf2 mRNA levels

**Figure 7:**
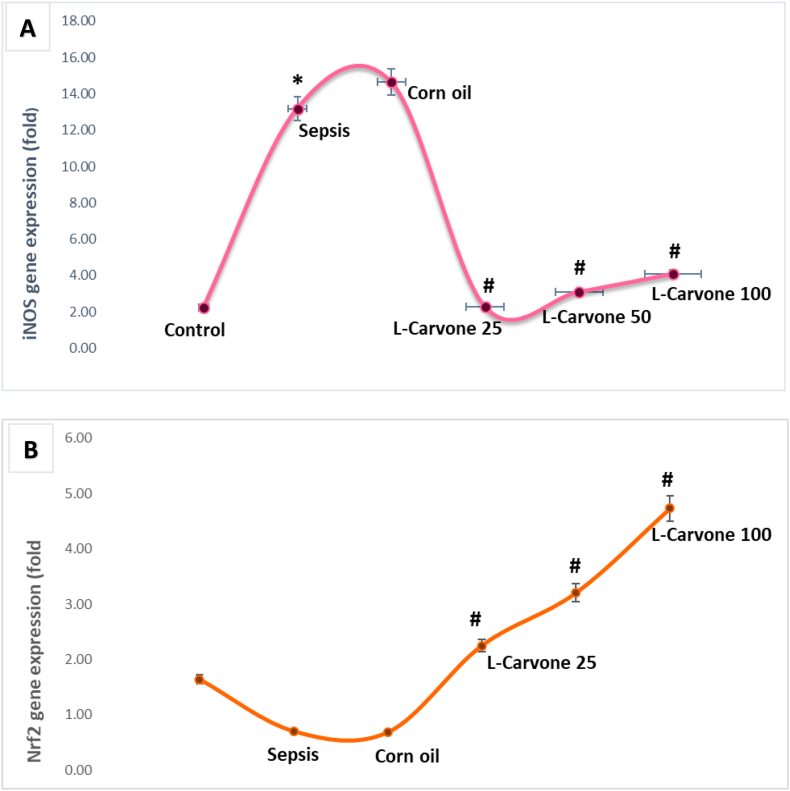
**Influence of the investigated agents on iNOS and Nrf2 gene expression**. **A.** Transcriptional profile of iNOS **B**. Transcriptional profile of Nrf2. Results are means ± SEM; ∗P < 0.05 compared with control, #P < 0.05 compared with sepsis/induction.

Markedly greater inducible nitric oxide synthase (iNOS) expression was observed in the sepsis and corn oil groups than the untreated control group (Figure 7A). Administration of LPS in the sepsis group, as well as solvent treatment in the corn oil group, resulted in substantial increases (approximately 13-fold) in iNOS levels, thus confirming the potent stimulation of the inflammatory nitric oxide pathway during SA-AKI. In contrast, L-carvone therapy at all three concentrations markedly decreased iNOS expression: the expression levels in all L-carvone groups were close to those in the healthy controls, thereby indicating a robust inhibitory effect on iNOS gene activation. These findings indicated that L-carvone markedly inhibits LPS-evoked iNOS upregulation, thereby decreasing excess nitric oxide generation and ROS formation, and contributing to the observed nephroprotective effects.

The gene expression of erythroid-associated nuclear transcription factor 2 (Nrf2) was markedly lower in the sepsis model than the untreated controls (P < 0.05) (Figure 7B), thus indicating suppression of the antioxidant defense system during SA-AKI. An identical decrease in the corn oil group confirmed that the solvent had no effect on Nrf2 expression. In contrast, intervention with L-carvone markedly restored and activated Nrf2 expression in a dose-dependent manner. At a dose of 25 mg/kg, Nrf2 expression was moderately greater than observed in the sepsis group (P < 0.05), and more pronounced upregulation was observed with 50 and 100 mg/kg doses.

### Influence of the investigated agents on apoptosis markers

**Figure 8:**
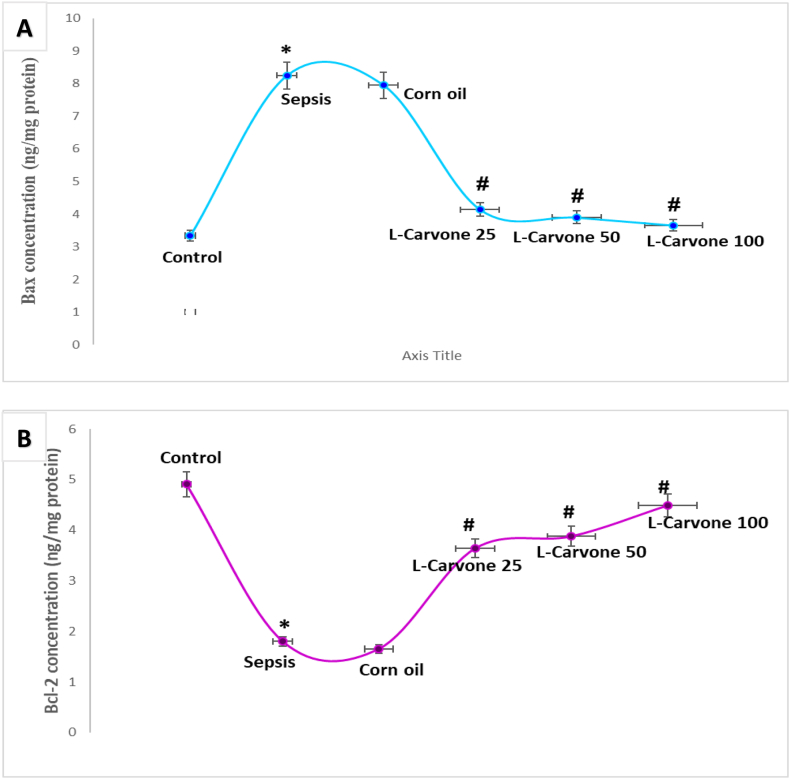
**Influence of the investigated agents on apoptotic and anti-apoptotic proteins (Bax and Bcl-2). A.** Influence of the investigated agents on Bax levels. **B.** Influence of the investigated agents on Bcl-2 levels. Results are means ± SEM; ∗P < 0.05 compared with control, #P < 0.05 compared with sepsis/induction.

The sepsis group exhibited markedly higher pro-apoptotic Bax and lower anti-apoptotic Bcl-2 levels in kidney tissues than the untreated control group (P < 0.05). The corn oil group similarly presented no notable differences with respect to the sepsis model (P > 0.05), thus indicating that the solvent had no influence on apoptosis-related markers. Intervention with L-carvone at all three doses resulted in markedly lower Bax and higher Bcl-2 renal levels than observed in the sepsis model (P < 0.05) (Figures 8A and **8B**).

### Influence of the investigated agents on oxidative stress markers

**Figure 9:**
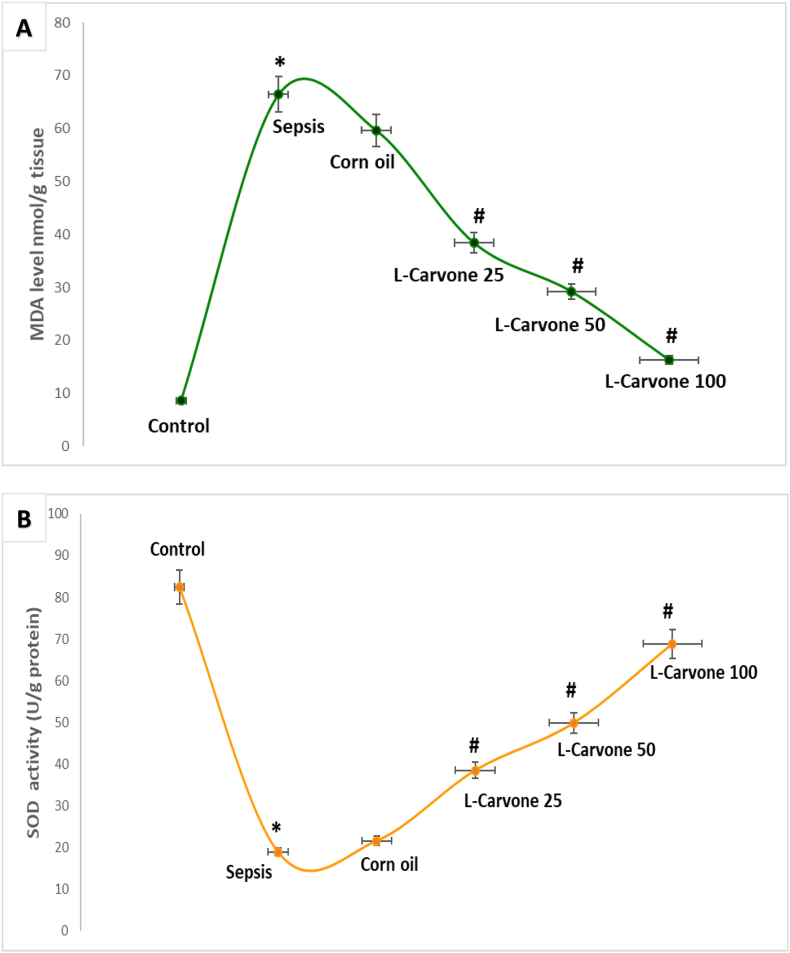
**Influence of the investigated agents on oxidative and anti-oxidative indicators (MDA and SOD). A.** Influence of the investigated agents on MDA levels. **B.** Influence of the investigated agents on SOD levels. Results are means ± SEM; ∗P < 0.05 compared with control, #P < 0.05 compared with sepsis/induction.

Mice challenged with LPS exhibited major oxidative imbalance, revealed by substantially higher MDA levels and lower SOD activity than observed in non-treated controls (P < 0.05). Similarly, the absence of substantial differences between the corn oil group and the sepsis model (P > 0.05) demonstrated that the solvent did not affect oxidative stress bio-indicators. Meanwhile, the L-carvone therapeutic intervention groups at all three administered doses, in contrast to the sepsis group, showed effective mitigation of these alterations, markedly diminished MDA concentrations, and restoration of SOD activity (P < 0.05) (Figures 9A and **9B**).

## Discussion

Sepsis is a deadly medical syndrome involving diverse functional and metabolic complications.^54^ Sepsis-exacerbated AKI leads to deterioration through inflammatory mechanisms.^22^^,^^55^ Conventional therapies for SA-AKI require prompt antibiotic delivery, appropriate vasopressor medications, and fluid supplementation. Unfortunately, because the existing treatment approach is nonspecific and ineffective, no efficient approach is available to avoid this deadly illness.^56^ Consequently, the discovery of innovative, successful medicines for septic renal impairment would have major therapeutic implications. Therapeutic drugs capable of arresting the progression of LPS-evoked SA-AKI must urgently be investigated. Moreover, the exact functions of L-carvone in the pathogenic process of LPS-aggravated SA-AKI remain to be explored.

Herein, we created a mouse model of sepsis-related renal impairment via LPS intraperitoneal administration. In line with earlier reports, the use of 10 mg/kg LPS produced kidney tissue destruction, as evidenced by elevated urea and creatinine, as well as substantial renopathological abnormalities including tubular epithelial cell detachment, absence of the brush boundary integrity, and cortical tubule widening.^37^ Our findings demonstrated that L-carvone at low (25), moderate (50), and high (100) mg/kg doses alleviated the creatinine and urea levels, corrected the histological modifications observed in the proximal tubules in LPS-challenged animals, and minimized the tubular injury scores. These preventive actions might have been partly due to amelioration of LPS-evoked augmentation of inflammation-associated mediators, specifically IL-1β and TNF-α.^29^^,^^30^ TNF-α is closely correlated with substantial tubular impairment, and IL-1β has likewise been found to be critical for the pathology of kidney deterioration.^20^^,^^57^^,^^58^ Suppression of these inflammatory components can safeguard the kidneys against damage initiated by LPS. Current studies have focused on identifying biochemical markers such as KIM-1 as a potential early and specific measure for renal destruction.^59^ Overproduction of KIM-1 after LPS challenge was demonstrated in this work and in other research.^60^ Our findings indicated that pre-administration of L-carvone at every tested dose suppressed KIM-1 levels in kidney homogenates. Furthermore, the secretion of KIM-1 by destroyed kidney cells has been found to activate regulatory components that govern the synthesis of chemotactic cytokines.^61^

TLR4 is highly expressed in immune cells, glomeruli in both podocytes and mesangial cells, and particularly the tubular epithelium. Its expression dynamically responds to LPS. TLR-4 translation appears to be unpredictable; at minimal baseline levels, it increases in response to LPS.^23^ Our findings revealed considerable TLR4 receptor elevation in the LPS model group after 24 h but not in non-treated controls, in agreement with previous study findings.^62^ Moreover, L-carvone at all three doses caused substantial declines in TLR4 production, thus suggesting preventive effects against AKI. We further examined signaling downstream of TLR4 to demonstrate the anti-inflammatory effects of L-carvone. TLR4 signaling after LPS binding can be categorized into MyD88-related and MyD88-unrelated pathways.^63^ NF-κB and its downstream cytokines that promote inflammation expression are governed by the MyD88-related pathway. NF-κB, a transcription factor with key roles in exacerbating inflammation,^64^ initiates the expression of numerous inflammatory genes, particularly IL-β and TNF-α, which have key functions in mediating inflammatory cascades.^65^, ^66^, ^67^ Another transcription factor activated via the MYD-dependent pathway is AP-1, which is essential for inflammation and controls the synthesis of these inflammatory mediators. Our findings demonstrated that NF-κB signaling was triggered in the kidneys of rodents injected with LPS, whereas pre-treatment with L-carvone considerably attenuated both these transcription factors, in contrast to observations in the LPS group. Interestingly, further attenuation of the levels of these transcription factors was observed with a 100 mg/kg dose. All doses of carvone substantially ameliorated proinflammatory cytokine amounts (IL-1β gene expression and TNF-α levels), in contrast to the sepsis/induction model. These findings were consistent with those from another investigation revealing that L-carvone doses of 50 and 100 mg/kg markedly decreased TNF-α amounts in irinotecan-aggravated intestinal damage in mice.^68^

Whereas the 25 mg/kg dose achieved at least moderate improvements in biochemical parameters, the 100 mg/kg dose led to greater attenuation of renal injury and cytokine release, thereby indicating dose-dependent renoprotective effects. Consequently, the protective potential of L-carvone was enhanced as the dose increased to 100 mg/kg, probably because of greater interaction with its molecular targets and increased antioxidant activity.^35^

Beyond its well-documented anti-inflammatory and antioxidative capabilities, D-carvone has achieved remarkable protective effects in some experimental models of renal damage. D-carvone markedly ameliorates lithium-driven nephrotoxicity, as evidenced by diminished kidney deterioration and histological transformation, suppression of oxidative stress, restoration of detoxifying functions, and promotion of Nrf2/HO-1 signaling transduction.^69^ In another model of renal injury caused by ischemia-reperfusion, D-carvone has been observed to mitigate kidney damage by inhibiting lipid peroxidation, restoring the apoptotic protein balance, and strengthening antioxidative defense systems, while preserving renal histology and function.^70^ Overall, these findings demonstrate that L-carvone delivers broad-spectrum medicinal advantages to the kidneys, presumably because of its prospective roles in regulating oxidative and inflammatory cascades, redox equilibrium enhancement, and cellular integrity repair. The current results in LPS-mediated renal sepsis are consistent with those from earlier studies implying that D-carvone provides considerable protection against a variety of nephrotoxic exposures via a coordinated antioxidant and antiinflammatory mechanism.^34^^,^^69^^,^^71^

Immediate TLR4 stimulation (MyD88-facilitated axis) leads to endosomal signaling, thus triggering NF-κB amplification and IFN-γ production.^72^^,^^73^ When IRF3 is phosphorylated and transported to the nucleus, it initiates the TRIF-associated route. This process promotes type I interferon generation and NF-κB signaling, and contributes to enhanced expression of inflammation-related cytokine genes.^74^ Our data revealed that the LPS group showed considerably greater IRF3 transcription at the kidney organ level than observed in non-treated controls. These findings are consistent with current research indicating that LPS administration leads to considerable IRF3 expression.^75^ In our study, low, moderate, and high doses of L-carvone resulted in markedly lower IRF3 mRNA levels than observed in the sepsis group, thus confirming L-carvone's suppressive effects on late-stage NF-κB and type I IFN transcription.

L-carvone appears to have nephroprotective effects through modulating TLR4-driven mechanisms and their downstream networks.^32^^,^^76^ Septic renal damage stimulates TLR4 and intermediate elements, i.e., MyD88 and TRIF, thus increasing the transcription factors NF-κB and AP-1.^12^ and further driving the formation of numerous inflammatory substances, and increasing renal inflammation severity. L-carvone disrupts the NF-κB-TLR4–MyD88 activation cascade by blocking IκBα decomposition and nuclear relocation of the NF-κB p65 monomer.^8^ Blocking NF-κB function decreases the inflammatory markers IL-1β, IL-6, IL-8, and TNF-α, and limits the generation of COX-2 and iNOS.^77^, ^78^, ^79^ The disruption of messenger proteins comprising p38, JNK, and ERK across the MAPK system minimizes AP-1-driven translation of pro-inflammatory gene products,^80^ and results in diminished oxidative damage and leukocyte migration in kidney tissues.

The degeneration and focal necrosis observed in the LPS group are known as downstream processes resulting from inflammation-driven oxidative and apoptotic attacks. The renoprotective effect of L-carvone is therefore due to inhibition of inflammatory signaling and consequent indirect decreases in cellular breakdown and death. However, we did not directly examine apoptotic markers (such as caspase-3 expression or TUNEL). Further analysis is therefore necessary to study the underlying mechanism linking L-carvone's anti-inflammatory effect and potential anti-apoptotic functions in renal tissues.

Our results revealed that application of L-carvone to septic LPS-challenged mice markedly mitigated iNOS expression in renal tissue. Therefore, L-carvone might prevent NO overproduction, and subsequently restrict peroxynitrite formation and oxidative attack during sepsis.^81^ This finding is consistent with findings from prior reports indicating that L-carvone has anti-inflammatory and antioxidant properties by inhibiting inflammatory factors and immune-related enzymes, namely iNOS and COX-2, thereby counteracting nitric oxide accumulation and ameliorating histological irregularities.^32^ Furthermore, L-carvone has been demonstrated to inhibit LPS-exacerbated phagocytic activation by interfering with the MAPK and NF-κB pathways, which are key regulators of iNOS expression.^34^ Together, these data suggest that L-carvone modifies the TLR4/NF-κB/iNOS/NO axis,^82^^,^^83^ thus restoring redox balance and renal micro-dynamics during sepsis. Accordingly, our study supports a hypothesis in which the sepsis-protective effects of L-carvone in the LPS-challenged sepsis model are partly attributable to its inhibition of iNOS hyper-regulation and the consequent attenuation of inflammatory and nitrosative pathways, in agreement with the mechanistic framework observed in various models of inflammation-linked conditions.^36^^,^^71^^,^^84^^,^^85^

Moreover, we observed that treating LPS-challenged mice with L-carvone markedly alleviated the expression of the transcriptional element Nrf2 in kidney tissue in a dose-dependent manner. L-carvone promotes the Nrf2-based antioxidative defensive process, an essential counter-regulatory mechanism against oxidative attack.^31^^,^^33^ Stimulation of Nrf2 leads to generation of an array of detoxifying enzymes, notably HO-1, GPx, SOD, and catalase.^86^ Nrf2 activation optimizes redox equilibrium and indirectly hinders NF-κB signaling through inhibition of intracellular ROS.^87^^,^^88^

The sepsis group displayed a marked decline in Nrf2 expression, reflecting a weakened antioxidant defense system under septic conditions.^89^ Restoration of Nrf2 expression mediated by L-carvone suggested that this compound activates the Nrf2/ARE pathway, thereby enhancing redox balance and strengthening cellular resistance to oxidative damage. These findings are consistent with those from previous reports showing that L-carvone and other monoterpenes can increase Nrf2 and HO-1 expression in various models of inflammation and oxidative harm.^31^^,^^36^ Ulas et al. have demonstrated that L-carvone increases Nrf2 and HO-1 content in a model of LPS-aggravated pulmonary impairment, and these findings are associated with compromised tissue structure and decreased lipid peroxidation.^34^ The subsequent decline in oxidative and inflammatory stress preserves mitochondrial polarization, and consequently impedes cytochrome *c* liberation and caspase signaling.^90^

Together, these data indicate that L-carvone might have a dual regulatory effect, by activating the Nrf2 pathway responsible for antioxidant defense and inhibiting the inflammatory NF-κB/iNOS pathways, thus restoring the equilibrium between oxidative and immune-related mechanisms in septic kidneys. In addition, Nrf2 activation might contribute to microvascular protection and mitochondrial stability, by preventing cytochrome *c* release and caspase activation,^90^ key mechanisms that preserve renal blood flow and function during septic injury.

Notably, we found that L-carvone administration in septic mice exposed to LPS markedly decreased the levels of apoptotic proteins such as Bax while simultaneously increasing levels of the anti-apoptotic bio-marker Bcl-2 in kidney tissues. In line with these outcomes, L-carvone has been established to increase the synthesis of anti-apoptotic bio-indicator (Bcl-2) and inhibit pro-apoptotic regulators (Bax), thus implying substantial inhibition in apoptosis in kidney tissues.^82^^,^^91^ Additionally, the modulatory activity toward the autophagic markers LC3 and p62 promotes restoration of autophagic transport, which might contribute to cellular adaptability in stress settings.^92^^,^^93^

Our study revealed that L-carvone treatment effectively ameliorated oxidative stress in the kidneys of mice undergoing LPS-triggered sepsis, as reflected by a substantial decline in MDA production and restoration of SOD activity. The augmented MDA levels in the sepsis group suggested extensive lipid peroxidation, which is an important characteristic of oxidative injury provoked by excessive generation of ROS during the course of sepsis.^22^^,^^94^ This oxidative insult disrupts cellular barrier function and is involved in the progression of kidney impairment, as previously described for LPS-driven sepsis models.^20^^,^^37^ Similarly, the diminished SOD activity observed in septic kidneys suggests an impaired antioxidative defense system and promotes vulnerability of kidney tissues to ROS-induced injury.^95^^,^^96^ SOD has an essential role as an enzymatic scavenger of powerful superoxide radicals, whereas SOD deficiency worsens oxidant harm and enhances inflammation^97^, ^98^, ^99^

We observed that L-carvone treatment substantially reversed these pathological alterations. The strong decline in MDA levels after L-carvone administration might indicate that this monoterpene inhibits peroxidation process and consequently prevents ROS damage to cellular membranes.^100^^,^^101^ This antioxidative property is consistent with earlier reports showing the free radical-scavenging ability of L-carvone in multiple experimental models.^35^^,^^102^ Additionally, the elevated SOD activity in the carvone-treated groups might require the endogenous antioxidative defense in kidney cells to scavenge superoxide radicals and ameliorate oxidative attack.^103^^,^^104^ This combined action might potentially be responsible for renoprotection, as demonstrated by our research. The antioxidant effects of L-carvone might stem from enhancement of the Nrf2 axis and consequently enhance the expression of many antioxidative elements, including SOD and catalase.^31^^,^^69^ Moreover, by hindering ROS generation, L-carvone might indirectly inhibit subsequent inflammatory cascades, given that oxidative attack and inflammation are closely correlated in the pathophysiology of sepsis.^90^^,^^105^ Together, our data suggested that L-carvone attenuates oxidative stress and restores the renal redox state, and therefore might serve as a treatment for kidney injury in septic conditions.

**Figure 10:**
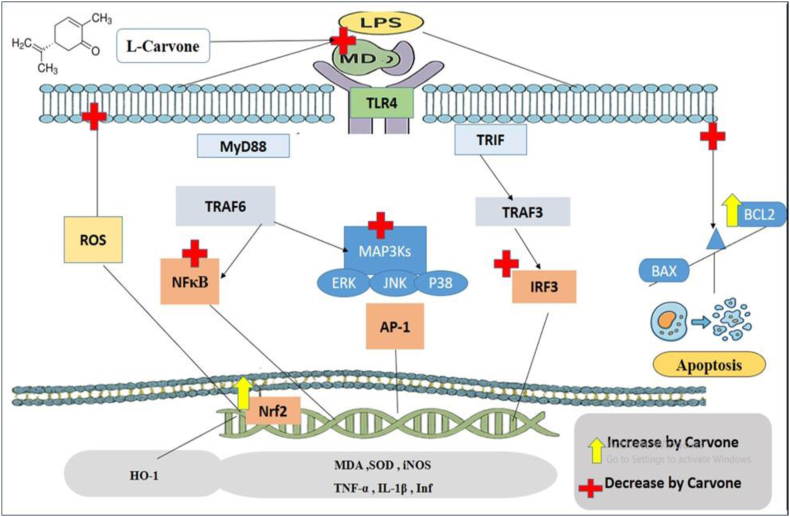
Graphical depiction of the effects of L-carvone in LPS-evoked renal sepsis.

These preventive mechanisms together maintain renal integrity and function by preventing oxidative harm, inflammation, necrosis, and apoptosis. L-carvone appears to operate by combating TLR4-mediated inflammation and activating the Nrf2/HO-1 antioxidant signaling pathway. This synchronized interplay of anti-inflammatory and antioxidant actions is consistent with previous findings in liver and lung inflammation models indicating that L-carvone is a possible cytoprotective treatment against septic kidney damage.^32^^,^^36^ Figure 10 provides a graphical depiction of the effects of L-carvone in LPS-evoked renal sepsis.

### Study limitations

The investigation involved a limited number of mice, thus potentially restricting the generalizability of the results. In addition, the analysis of only a single post-treatment time-point restricted the understanding of temporal changes in renal recovery and inflammation. Moreover, the results were derived from a preclinical in vivo animal model, and their translation to human sepsis-associated renal injury remains to be validated. Additional biochemical markers and signaling pathways involved in renal protection were not explored.

Although our results illustrated a preventive role of L-carvone in vivo, we did not verify these effects at the protein level, because doing so was beyond the scope of our research funding. Subsequent investigations to verify regulation of TLR4-mediated signaling should be performed with western blotting for NF-κB/TLR4/AP-1/IRF3 pathways and the iNOS/Nrf2 axes. Moreover, in vitro validation in renal tubular epithelial cell models could further identify the mechanisms involved inhibition of TLR4-mediated inflammatory pathways and the direct cytoprotective action of L-carvone at the cellular level. Additionally, this research examined TLR4 and NF-κB expression but did not specifically measure MyD88 levels. Our interpretations of MyD88-independent NF-κB activation were based on documented mechanisms and should be viewed with caution. In addition, although our data provide morphological evidence of renal protection by L-carvone and its modulatory effects toward key parameters in the apoptotic response (Bax and Bcl-2), the association between diminished inflammation and prevention of cell death requires investigation in additional studies. Further use of specific assays such as caspase-3 activity and TUNEL staining would support the anti-apoptosis benefit of L-carvone in sepsis-related kidney impairment. Finally, more thorough analysis of interstitial nephritis and cellular infiltration is needed to provide a better overview of renal injury.

## Conclusion

This work demonstrated that L-carvone confers significant renoprotective effects against LPS-evoked sepsis-driven kidney impairment. L-carvone efficiently mitigated oxidative injury, inflammation, and apoptosis in kidney tissues by blocking TLR4/NF-κB/AP-1/IRF-3 inflammatory signaling and iNOS-driven NO overproduction, while concomitantly stimulating Nrf2 antioxidant mechanisms. Those biochemical improvements were validated by marked decreases in KIM-1, BUN, and creatinine, as well as substantial reversal of kidney histopathological alterations. Overall, this study suggested that L-carvone is a potential therapeutic candidate for attenuating sepsis-aggravated kidney deterioration. However, additional research on dose adjustment and clinical translational investigations is necessary.

## Ethics approval

The study proposal design was validated by the Institutional Review Board (IRB) of the University of Baghdad's College of Pharmacy (permission number 202404466). All experiments adhered to institutional and national standards for the care and administration of experimental animals. The IACUC/Ethical Committee granted ethical authorization.

## Authors contributions

SMS took part in revising and preparing the draft version of the manuscript, collaborating in the experimental planning of the study, and securing funding and other support. SHK participated in resources for the research, assisted in supervision and validation, and approved the final version of the manuscript. Haider Ridha-Salman performed an extensive evaluation of the manuscript, by offering input and critiques, organizing and compiling data, integrating methodological strategies, and providing logistical assistance.

All authors have critically reviewed and approved the final draft and are responsible for the content and similarity index of the manuscript.

## Availability of data

Data validating the results of this research can be obtained from the corresponding author on reasonable request.

## Source of funding

This work was performed without any external funding.

## Conflict of interest

The authors have no competing interests to disclose.
